# A Case of Worsened Refractory Ascites due to Prednisolone Administration for Stricture Prevention after Endoscopic Submucosal Dissection for Extensive Early Esophageal Cancer: Case Report and Literature Review

**DOI:** 10.1002/deo2.70222

**Published:** 2025-10-15

**Authors:** Yuki Tamura, Masanori Sekiguchi, Kaho Honda, Yu Maruyama, Kenta Ito, Makiko Inoue, Mitsuhiko Shibasaki, Daichi Takizawa, Hirotaka Arai, Toshio Uraoka

**Affiliations:** ^1^ Department of Gastroenterology Maebashi Red Cross Hospital Gunma Japan; ^2^ Department of Gastroenterology and Hepatology Gunma University Graduate School of Medicine Gunma Japan

**Keywords:** decompensated cirrhosis, ESD, prednisolone, refractory ascites, SESCC

## Abstract

Endoscopic submucosal dissection (ESD) is widely used for early esophageal cancer, even in patients with liver cirrhosis (LC). Corticosteroids, administered orally or by local injection, are often used to prevent post‐ESD esophageal stricture. However, their safety in patients with decompensated LC and refractory ascites remains unclear. A man in his 70s with alcohol‐related decompensated LC and refractory ascites underwent ESD for subcircumferential superficial esophageal squamous cell carcinoma located on esophageal varices. To prevent post‐ESD stricture, both oral prednisolone and local triamcinolone were administered. Ascites worsened significantly, and large‐volume paracentesis was performed. Subsequently, the patient developed a mural thrombus in the superior mesenteric vein and non‐occlusive mesenteric ischemia, leading to bowel perforation and death on day 51 post‐ESD. In LC patients with refractory ascites, oral corticosteroids may exacerbate ascites and increase thrombotic risk, potentially leading to fatal complications. This case highlights the need for careful risk–benefit assessment of subcircumferential ESD in vulnerable cirrhotic patients.

## 1 Introduction

Endoscopic submucosal dissection (ESD) is a widely accepted treatment for early‐stage esophageal cancer confined to the mucosa. Patients with liver cirrhosis (LC), particularly those with alcohol‐related etiology, are at increased risk of developing esophageal cancer [1]. Several studies have demonstrated the safety and high R0 resection rates of ESD in patients with LC, including those with decompensated liver function, although with limited follow‐up periods [2].

Esophageal varices (EVs) are a common and significant complication in patients with LC. Recently, ESD has been performed for early esophageal cancers located adjacent to or on EVs, often after endoscopic variceal ligation (EVL) [3]. Although ESD has enabled en bloc resection of large lesions, post‐ESD esophageal strictures remain a major concern, especially after extensive circumferential resections. To prevent stricture formation, oral administration and/or local injection of corticosteroids are commonly employed [4].

To our knowledge, no reports have described ESD for extensive early esophageal cancer requiring stricture prevention in a patient with LC complicated by refractory ascites and EVs. In the present case, we administered both oral prednisolone and local triamcinolone injection to prevent post‐ESD stricture. Subsequently, the patient developed a mural thrombus in the superior mesenteric vein (SMV), along with marked worsening of ascites. Eventually, he died from intestinal perforation caused by non‐occlusive mesenteric ischemia (NOMI) and complete SMV obstruction. Systemic corticosteroids likely contributed to thrombus formation and fluid retention, exacerbating refractory ascites.

We herein report this rare and clinically significant case, together with a review of the relevant literature.

## 2 Case Report

**FIGURE 1:**
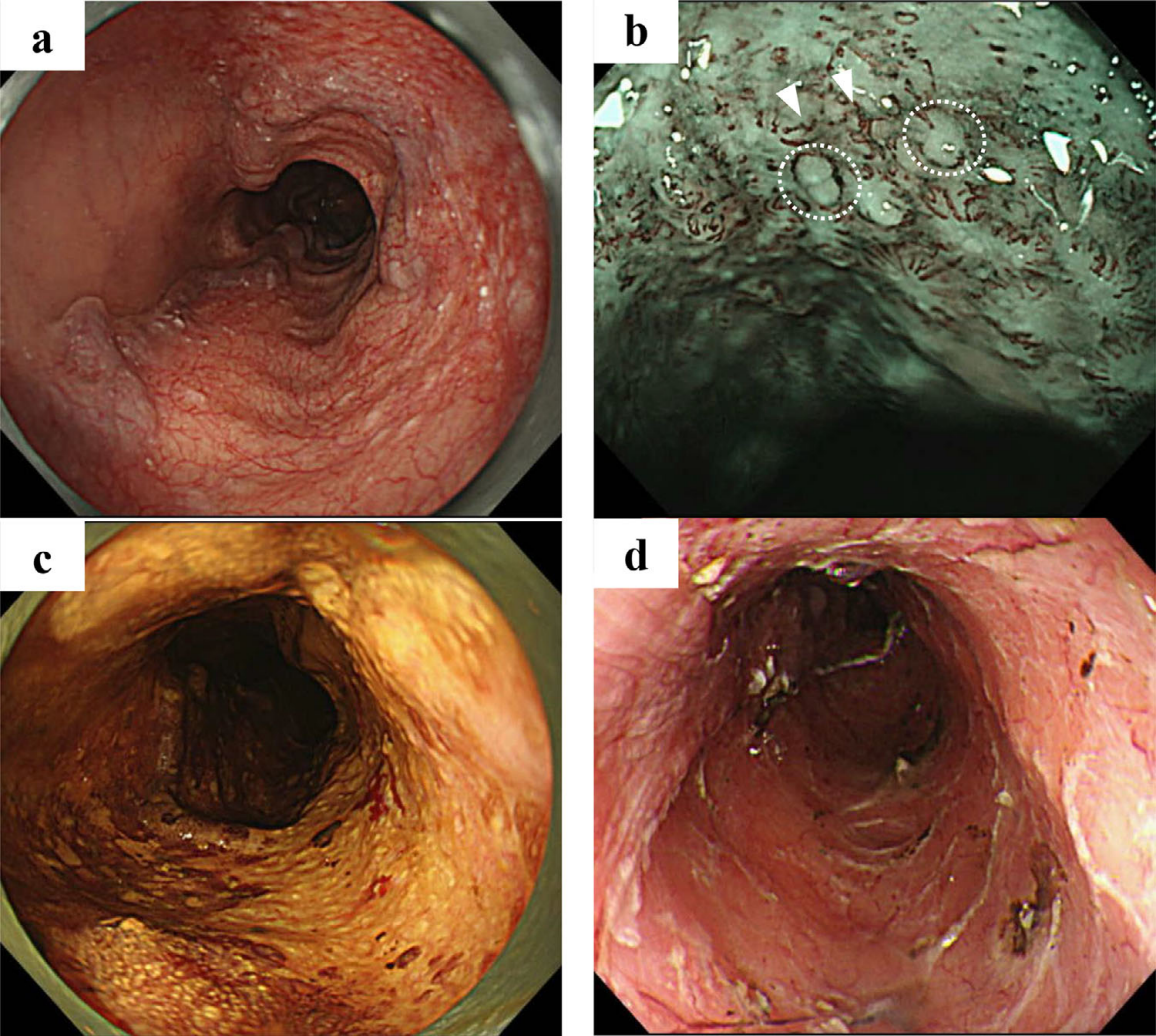
Subcircumferential superficial esophageal squamous cell carcinoma (SESCC) was detected in the cervical to upper thoracic esophagus. The lesion was located on F2‐grade EVs (a, c). Abnormal microvessels without a loop‐like formation were presented (b, arrowhead), and small‐sized avascular areas were shown by dotted lines. Endoscopic submucosal dissection (ESD) was performed, resulting in a subcircumferential post‐ESD ulcer (d).

A man in his 70s had been regularly followed at our hospital for decompensated LC secondary to alcohol consumption. Approximately 2 years earlier, he began receiving cell‐free and concentrated ascites reinfusion therapy (CART) every 1–2 months to manage refractory ascites. During routine annual esophagogastroduodenoscopy (EGD), superficial esophageal squamous cell carcinoma (SESCC) was detected in the cervical to upper thoracic esophagus. The lesion was located on F2‐grade EVs and had spread in a subcircumferential manner (Figure 1a,c). Narrow‐band imaging revealed type B2 vessels without loop‐like formation and a small avascular area, based on the Japan Esophageal Society classification (Figure 1b). The tumor was classified as T1a (invasion into the muscularis mucosae), and ESD was considered a relative indication for treatment [5].

The patient's height, weight, and body mass index (BMI) were 164 cm, 51.5 kg, and 19.4 kg/m^2^, respectively. He had ceased alcohol consumption 5 years prior, but continued smoking 20 cigarettes per day. Laboratory tests showed thrombocytopenia (platelet count: 8.7 × 10^4^/µL) and hypoalbuminemia (albumin: 3.2 g/dL), but no jaundice (total bilirubin: 1.0 mg/dL) or coagulopathy (prothrombin time: 89%). Other results included hemoglobin 14.5 g/dL, leukocyte count 6400 /µL, creatinine 1.1 mg/dL, and serum ammonia 18 µg/dL. He was receiving three types of diuretics—furosemide, spironolactone, and tolvaptan—for refractory ascites. He had no history of hepatic encephalopathy. Systemic computed tomography (CT) revealed no evidence of hepatocellular carcinoma or metastasis of esophageal cancer.

To reduce the risk of bleeding during ESD, EVL was performed in advance. Five variceal bands were applied beginning at the gastroesophageal junction and extending proximally. Because of insufficient eradication, two additional bands were placed approximately 1 cm distal to the tumor. EVL was well tolerated, with no worsening of ascites, and laboratory parameters remained stable.

One month later, the patient was hospitalized for ESD. Multiple episodes of transient bleeding occurred from sites that were difficult to coagulate, but the procedure was completed as an en bloc resection over 225 min without significant blood loss. Because the post‐ESD ulcer was subcircumferential (Figure 1d), local injection of triamcinolone acetonide was performed to prevent stricture formation.

Histopathological examination revealed well‐ to moderately‐differentiated squamous cell carcinoma invading the muscularis mucosae (pT1a‐MM). Although the resection margin was negative, lymphatic invasion was observed (Ly1), and the procedure was thus deemed non‐curative.

**FIGURE 2:**
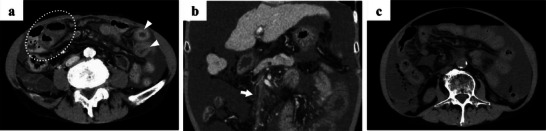
(a) The arterial phase of contrast‐enhanced computed tomography (CT) image demonstrates the profound contrast difference between the normal (arrowhead) and hypoenhancing ischemic (dotted line) loops. (b) Venous phase contrast‐enhanced CT reveals extensive mesenteric venous thrombosis (arrow) with a large volume of ascites. (c) CT demonstrates intraperitoneal free air, although the perforation site was not clearly identified.

**FIGURE 3:**
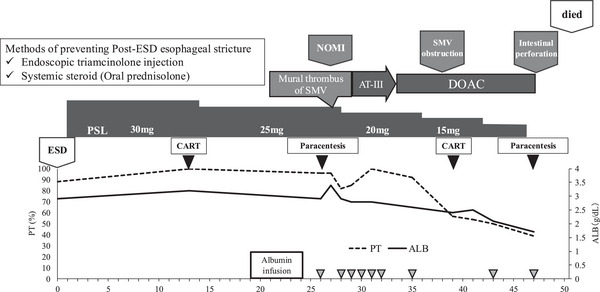
ESD, endoscopic submucosal dissection; NOMI, non–occlusive mesenteric ischemia; SMV, superior mesenteric vein; AT–III, antithrombin III; DOAC, direct oral anticoagulants; PSL, prednisolone; CART, cell–free and concentrated ascites reinfusion therapy; PT, prothrombin time; ALB, albumin. Clinical course after ESD. Both local triamcinolone injection and oral prednisolone were administered to prevent post–ESD esophageal stricture. Liver function remained stable except for worsening ascites by day 26. Following the onset of NOMI, liver function progressively deteriorated, requiring repeated paracentesis and albumin infusion.

Postoperatively, the patient did not develop anemia or coagulopathy requiring transfusion. Oral prednisolone 30 mg/day was initiated on postoperative day 2 for stricture prevention and tapered 5 mg per 1–2 week. While hypoalbuminemia did not worsen, the ascites markedly increased. However, the polymorphonuclear leukocyte count in the ascitic fluid did not exceed 250 cells/mm^3^, indicating no evidence of spontaneous bacterial peritonitis (Table 1). CART was performed on day 13, retrieving 3000 mL of ascitic fluid. The patient was discharged on day 15. However, on day 26, he presented without an appointment due to increased abdominal distension, and paracentesis yielded 6000 mL of ascitic fluid. At that time, 20 g of albumin was administered, which was considered insufficient. The next day, he returned with abdominal pain and exhibited generalized abdominal tenderness. Contrast‐enhanced CT revealed a mural thrombus in the SMV and partial bowel wall hypoenhancement (Figure 2a), without occlusion of the superior mesenteric artery (SMA), suggesting NOMI. Figure 3

**TABLE 1:** Ascitic fluid laboratory results after a marked increase in ascites following prednisolone administration.

Ascitic fluid characteristics	
Color	yellow
Clarity	clear
Specific gravity	1.005
Albumin	0.4 g/dL
Total white blood cell count	81 /µL
Polymorphonuclear cells	9.9%
Absolute polymorphonuclear cell count	8 /µL
Mononuclear cells	90.1%
Absolute mononuclear cell count	73 /µL
Serum‐ascites albumin gradient	3.2 g/dL

It was considered that the rapid loss of intravascular volume from large‐volume paracentesis had contributed to NOMI. Daily intravenous albumin was administered for volume support. As the antithrombin III (ATIII) level was below 70%, ATIII replacement was given for five days, but the mural thrombus persisted. Therefore, direct oral anticoagulants were started. By day 38 after ESD, complete SMV occlusion and progression of bowel ischemia were confirmed (Figure 2b). The patient and his family declined surgical intervention. Eventually, he developed intestinal perforation and died on day 51 post‐ESD (Figures 2, 3).

## 3 Discussion

Although the safety and efficacy of ESD for SESCC in patients with LC and EVs have been demonstrated in several studies, ESD for large SESCCs requiring stricture prevention in patients with decompensated cirrhosis and portal hypertension has not been previously reported.

Oral prednisolone and/or local injection of triamcinolone acetonide are commonly used to prevent post‐ESD esophageal stricture. A recent multicenter randomized controlled trial in Japan, which included 281 patients—43 of whom had lesions involving more than three‐quarters of the esophageal circumference—found that oral prednisolone was not superior to local steroid injection therapy [6]. However, the choice of treatment is typically left to the discretion of the attending physician. In our case, the risk of post‐ESD stricture was considered high because the resection area was large (52 × 43 mm) and involved the cervical esophagus. Therefore, we chose to administer both oral prednisolone and local triamcinolone injection.

The marked increase in ascites observed in this patient could not be explained by intraoperative fluid administration, as no fluid infusion was given between the first and second paracenteses. Liver function remained stable, except for worsening ascites. Based on these observations, we suspect that the administration of oral prednisolone contributed directly to the exacerbation of ascites.

Glucocorticoids are known to increase the risk of venous thromboembolism by promoting a procoagulant state [7]. In our case, a mural thrombus in the SMV may have formed after the prolonged ESD procedure, potentially due to intraoperative immobility, minor bleeding, and increased portal pressure following prior EVL [8]. The thrombus may have subsequently enlarged under the influence of systemic prednisolone, which is known to promote a procoagulant state. This likely worsened portal hypertension and contributed to the progression of refractory ascites.

In addition, prednisolone possesses mineralocorticoid activity (Table 2), promoting sodium reabsorption and potassium and hydrogen excretion in the renal tubules, thereby increasing extracellular fluid volume. This effect likely contributed to ascites [9].

**TABLE 2:** Relative potencies of representative glucocorticoids.

Compound	Glucocorticoid activity (relative)	Mineralocorticoid activity (relative)
Cortisol	1	1
Cortisone	0.8	0.8
Prednisolone	4	0.8
Methylprednisolone	5	0
Triamcinolone	5	0
Dexamethasone	25	0
Betamethasone	25	0

Glucocorticoid and mineralcorticoid activity of glucocorticoids, relative to cortisol.

The immediate cause of death was perforation due to acute mesenteric ischemia (AMI). AMI is typically categorized into three main types: arterial embolism or thrombosis, NOMI, and mesenteric venous thrombosis. Although a mural thrombus was observed in the SMV, it was not completely occlusive, and the diagnosis was NOMI. NOMI is caused by mesenteric hypoperfusion, which is often triggered by reduced cardiac output. In this case, large‐volume paracentesis was thought to be the precipitating factor [10].

In patients with LC complicated by refractory ascites, ESD for extensive early esophageal cancer requiring stricture prevention must be undertaken with great caution. In such patients, the use of oral prednisolone for post‐ESD stricture prevention may not be advisable due to the potential risks of fluid retention and thrombotic complications.

## Ethics Statement

The patient provided his written informed consent for the publication of the details of his case.

## Conflicts of Interest

One of the co‐authors, Professor Uraoka, serves as a Deputy Editor‐in‐Chief of DEN Open.
